# The Medical Law

**Published:** 1895-01

**Authors:** 


### *EDITORIAL.*

The office of THE JOURNAL is in room 330 Equitable Building.

Address all communications, and make all remittances payable to THE ATLANTA MEDICAL AND SURGICAL JOURNAL, Atlanta, Ga.

Articles for publication should reach this office not later than the 15th instant.

The editors are not responsible for views expressed by contributors.

### THE MEDICAL LAW.

By the time this reaches our readers our medical law will be in operation, having been passed unanimously by both branches of the legislature, signed by the Governor, and a board of examiners appointed. We believe that all honest and conscientious physicians in the State, regardless of the so-called schools, will be delighted at this result. Now, with good boards we may hope to keep out of the State any additional bunco-doctors and medical black-legs who may be casting about for a congenial abiding place. To all such Ohio, Maine, Massachusetts, New Hampshire, and Rhode Island are still fully open. We don't want them down here, and we believe that our boards will now admit to practice in this State only such physicians as give indications of being competent and worthy.

Following is the law as it now stands :

AN ACT

To establish Boards of Medical Examiners for the State of Georgia; to define their duties and powers; to protect the people from illegal and unqualified practitioners of medicine and surgery; to regulate the issuing and recording of licenses; to prescribe penalties for the violation of this act; and for other purposes.

SECTION 1. *Be it enacted by the General Assembly of Georgia, and it is hereby enacted by authority of the same,* That within thirty days after the passage of this Act, it shall be the duty of the Governor to appoint for this State three separate Boards of Medical Examiners of five members each, as follows: One board to consist of five members of the regular school of medicine; one board of five members of the eclectic school of medicine; and one board of five members of the homeopathic school of medicine. The members of each of said boards shall be men learned in medicine and surgery, of good moral and professional character, and graduates of reputable medical colleges; but none of them shall be members of the faculty of any medical college. Each of said three boards shall be wholly independent of and separate from the other two in the performance of the duties herein required of each of said boards. A majority of each board shall constitute a quorum.

SEC. 2. *Be it further enacted,* That the term of office of said members shall be for the term of three years; *provided,* that two members of each board shall first be appointed for one year, two for two years, and one for three years; and subsequently each appointment shall be for the full term of three years. Any vacancy that may occur in said board, in consequence of death, resignation, removal from the State, or from other cause, shall be filled for the unexpired term by the Governor.

SEC. 3. *Be it further enacted,* That immediately and before entering upon the duties of said office, the members of said Boards of Medical Examiners shall take the following oath: "I do swear that I will faithfully perform the duties of a member of the Board of Medical Examiners for the State of Georgia to the best of my ability, so help me, God," and shall file the same in the office of the Governor of the State, who, upon receiving the said oath of office, shall issue to each examiner a certificate of appointment.

SEC. 4. *Be it further enacted,* That immediately after the appointment and qualification of said members, each board shall meet and organize. The officers of said board shall be a president, vice-president, and secretary (who shall act as treasurer). Said officers shall be members of and elected by their respective boards. Each board shall hold two regular meetings in each year. One meeting shall be held at such time, on or just before graduation day of each medical college now chartered, or that may hereafter be chartered, in this State; and the Board of Examiners, after consultation with the faculty of said college, shall fix a time for its meeting to suit a majority of the students graduating from said college; the other on the second Tuesday in October. The first meeting shall be held in the city of Atlanta, and the succeeding meetings of each board may be held in such city as each board may determine for itself. Special meetings may be held upon the call of the president and two members of each board; but there shall not be less than two regular meetings in each year. Each board may prescribe rules, regulations, and by-laws for its proceedings and government. And each board shall examine and pass upon the qualifications of applicants for the practice of medicine in this State as herein provided.

SEC. 5. *Be it further enacted,* That it shall be the duty of each board, at any of its meetings, to examine only persons making application to it, who are graduates of an incorporated medical college, school, or university that requires not less than three full courses of study of six months each, who shall desire to commence the practice of medicine or surgery in the State, and who shall not by the provisions of this Act be exempt from such examination; but any person now matriculated as a student of medicine at any medical college, after graduation, and any person from another State who shall have graduated prior to April 1, 1895, at a lawfully chartered medical college, requiring only two full courses of study, shall be eligible for examination and license; *provided*, always, that the applicant for such examination shall hold a lawfully conferred diploma from an incorporated medical college which conforms to the system of practice represented by the board to which the application shall be made, unless the applicant desires to practice a different system from that recognized in his diploma; then he shall appear before the board which represents the system that he proposes to practice. But in no event shall an applicant who stands rejected by one of said boards be examined or licensed by either of the other boards. If the applicant desires to practice a system not represented by any of the boards hereby established, he may elect for himself the board before which he will appear for examination. When an applicant shall have passed an examination satisfactory as to proficiency before the board in session, the president thereof shall grant to such applicant a certificate to that effect. A fee of ten dollars shall be paid to such board, through such officer or member as it may designate, by each applicant before such examination is had. In case an applicant shall fail to pass a satisfactory examination before any board, he shall not be permitted to stand any further examination before any of the boards within the next three months thereafter. Nor shall he again have to pay the fee prescribed as aforesaid for any subsequent examination; *provided*, that when, in the opinion of the president of any board, any applicant has been prevented by good cause from appearing before said board, the president and two members of said board designated by him shall constitute a committee, who shall examine such applicant, and may, if they see fit, grant him a certificate which shall have the same force and effect as though granted by a full board, until the next regular meeting of the board, when, if the applicant fails to appear for examination, said certificate shall be void.

SEC. 6. *Be it further enacted*, That the fund raised from the fees aforesaid shall be applied by each examining board to the payment of its expenses and to making a reasonable compensation to the president, secretary, and members thereof.

SEC. 7. *Be it further enacted*, That before any person who obtains a certificate from any board, or from a committee of any board, may lawfully practice medicine or surgery in this State, he shall cause the said certificate to be recorded in the office of the clerk of the superior court in the county in which he resides. But, if he does not reside in the State of Georgia, he shall cause said certificate to be recorded in any county within this State in which he offers to practice. The certificate shall be recorded by the clerk in a book to be kept for that purpose. It shall be indexed in the name of the person to whom the certificate is granted. The clerk's fee for recording a certificate shall be the same as for recording a deed.

SEC. 8. *Be it further enacted*, That this Act shall take effect from and after the first day of January, 1895, and that it shall be unlawful thereafter for any person to commence the practice of medicine or surgery in this State without complying with the provisions of this Act. But nothing in this Act shall apply to persons now lawfully engaged in the practice of medicine or surgery in the State of Georgia, to any commissioned medical officer or contract surgeon of the United States army, or navy, or marine hospital service, in the performance of their duties as such, nor to any physician or surgeon residing in any State or Territory of the United States, or in the District of Columbia, who may be, *bona fide*, called in consultation in a special case with a physician or surgeon residing in this State; nor shall this Act be construed as affecting or changing, in any way, laws in reference to license tax to be paid by physicians and surgeons; *provided*, that a non-resident physician or surgeon, called in consultation in a special case as above prescribed, shall not be permitted to engage in continuous practice or consultation in connection with any resident physician or surgeon under any form of contract or agreement, direct or indirect.

SEC. 9. *Be it further enacted*, That any person shall be regarded as practicing medicine or surgery, within the meaning of this Act, who shall prescribe for the sick or those in need of medical or surgical aid, and shall charge or receive therefor money or other compensation or consideration, directly or indirectly; *provided*, however, that midwives and nurses shall not be regarded as practicing medicine or surgery.

SEC. 10. *Be it further enacted*, That any person who shall practice medicine or surgery in this State in violation of the provisions of this Act shall, upon conviction, be punished as prescribed in section 4310 of the Code of the State of Georgia for each offence; and it shall not be lawful for him to recover, by action, suit, motion, or warrant, any compensation for services which may be claimed to have been rendered by him as such physician or surgeon.

SEC. 11. *Be it further enacted*, That all laws and parts of laws in conflict with this Act be, and the same are, hereby repealed.

